# Eliminating circulating donor passenger leukocytes during clinical ex vivo lung perfusion does not attenuate inflammation

**DOI:** 10.1016/j.jhlto.2026.100619

**Published:** 2026-07-06

**Authors:** M.A. Hu, Z.L. Zhang, R.F. Hoffmann, C.T. Gan, E.A.M. Verschuuren, C. Van De Wauwer, H.G.D. Leuvenink, M.E. Erasmus

**Affiliations:** a Department of Cardiothoracic Surgery, University Medical Center Groningen, Groningen, Netherlands; b Department of Pulmonary Diseases and Lung Transplantation, University Medical Center Groningen, Groningen, Netherlands; c Department of Surgery, University Medical Center Groningen, Groningen, Netherlands

**Keywords:** Donor passenger leukocytes EVLP filtration

## Abstract

**Purpose:**

During Ex Vivo Lung Perfusion (EVLP), it is standard practice to incorporate a leukocyte filter to remove donor passenger leukocytes and attenuate their inflammatory effects. The widely used leukocyte filter, LeukoGuard 6, seems to be ineffective in reducing circulating leukocytes during EVLP. Therefore, we performed contemporary leukocyte filtration using BioR 02 plus leukocyte filters to reduce these leukocytes and investigate whether inflammation could be reduced.

**Methods:**

EVLP was performed for a minimum of 180 min with 15 bilateral donor lungs. Perfusate samples were taken from the in- and outlet of the leukocyte filter, which was subsequently clamped after 60 min. Contemporary parallel leukocyte filtration was performed in 9 EVLPs (intervention group) and in 6 EVLPs without (control group).

**Results:**

After EVLP, 6/6 donor lungs were transplanted in the control group and 6/9 in the intervention group. Leukocytes increased significantly from 30 to 180 min in the standard group compared with the intervention group. During EVLP IL-1, IL-6, IL-8, TNF-α, neutrophil elastase, CD-14, syndecan-1, hyaluronan and VCAM-1, levels were not significantly different between the groups at 90 and 180 min. Post-transplant, primary graft dysfunction (PGD) and 1-month survival were similar.

**Conclusion:**

The intervention group showed significantly lower circulating donor passenger leukocytes. However, this did not lead to attenuated inflammatory cytokines, leukocyte activation, glycocalyx shedding or endothelial activation. Clinical outcomes were similar between groups. Further research is needed to determine the exact effect of these donor passenger leukocytes.

## Introduction

Ex Vivo Lung Perfusion (EVLP) is an established method to expand the donor lung pool and offers a unique opportunity to reduce donor lung immunogenicity prior to transplantation.^1^, ^2^ It has been shown that EVLP influences donor leukocyte transfer and migration in the recipient.^3^ Consequently, EVLP can limit allorecognition and subsequent infiltration of recipient T cells into the allograft, both key processes in acute rejection.^3^

Donor leukocytes may impair lung function during EVLP.^4^ Therefore, eliminating these leukocytes could reduce inflammation and endothelial injury, thereby limiting Ischemia-Reperfusion Injury (IRI) and subsequent Primary Graft Dysfunction (PGD).^5^, ^6^ Endothelial injury can disrupt pulmonary homeostasis, as the endothelium and its glycocalyx layer are essential for lung function. Injured endothelium can induce edema development, which impairs lung function and contributes to declining donor lungs for transplantation.

Therefore, it is standardized to incorporate a leukocyte filter to reduce circulating donor passenger leukocytes during EVLP. The most commonly used leukocyte filter during EVLP is the Pall Leukoguard™ (LG6) filter, which is designed for extracorporeal use. However, postoperative results showed varying outcomes and evidence supporting its use during EVLP remains limited.^7^, ^8^, ^9^, ^10^, ^11^ A porcine study demonstrated that the LG6 only filters negligible amounts of leukocytes during EVLP due to rapid saturation.^12^ In contrast, rat EVLP performed using a different leukocyte filter significantly reduced inflammation and improved lung function.^13^

We observed high leukocyte counts despite the presence of an LG6 filter, suggesting its ineffectiveness in EVLP. Hence, we evaluated the effect of BioR 02 plus filters to reduce donor passenger leukocytes. We hypothesized that the number of circulating donor passenger leukocytes can be significantly reduced. This could potentially lead to decreased inflammation and endothelial injury during EVLP, ultimately leading to improved lung function, attenuated IRI and reduced risk of PGD development.^14^

## Materials and methods

### Study groups

**Figure 1:**
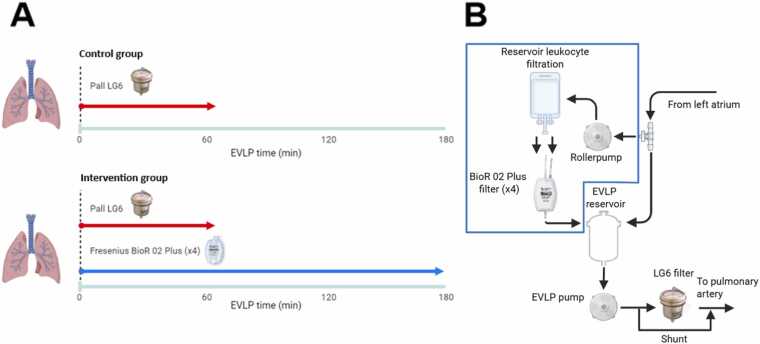
(A) Timeline overview of the control (top) and intervention group (bottom). In both groups the LG6 filter (red line) was clamped after 60 min of perfusion and perfusion was continued over a shunt. The intervention group underwent continuously leukocyte filtration from the start of EVLP (blue line). Donor lungs were either accepted for LTx or EVLP was continued after 180 min (B) Schematic overview of the filtration setup (blue bracket), consisting of a rollerpump, a reservoir and 4x BioR 02 Plus filters. The inlet of the system is connected to the line from the left atrium, while the outlet is connected to the EVLP reservoir.

Bilateral donor lungs underwent EVLP for a minimum of 180 min. In all EVLP procedures from May 2023 to May 2024, the LG6 filter was clamped from circulation after one hour of perfusion. Subsequently, perfusion was continued over a shunt to bypass the LG6. The first group received only leukocyte filtration within the first hour by the LG6 (control group). The second group underwent EVLP with contemporary leukocyte filtration from the start of EVLP (intervention group) (Figure 1A). This study was performed to evaluate contemporary leukocyte filtration during EVLP as part of standard clinical practice. Procedures were non-randomized and conducted sequentially in the order of the groups.

### Inclusion and exclusion criteria

Bilateral donor lungs with both logistical and medical indications for EVLP were included. Recipients younger than 18 years were excluded.

Medical indications included:

− Donor arterial oxygen tension (PaO₂) < 40 kPa at FiO₂ 1.0 and positive end-expiratory pressure (PEEP) of 5 cmH₂O despite recruitment maneuvers.
− Presence of pulmonary edema, hemorrhage, contusion, or emboli.
− Extended-criteria DCD (eDCD) donor lungs, defined as an agonal phase exceeding 2 h and 15 min.
− Suspected impaired lung quality based on microbiological, bronchoscopic, radiographic, or macroscopic assessment.

Logistical indications included:

− Standard-criteria donor lungs.
− Inability to perform conventional lung transplantation due to operating room scheduling or logistical constraints.
− Anticipated cold ischemic time exceeding 6 h, necessitating extension of the total preservation time.

### Endpoints

The primary outcomes are the amount of circulating donor passenger leukocytes, inflammatory cytokines, glycocalyx shedding products, leukocyte activation and endothelial activation markers. The secondary endpoints are 1-month survival and PGD grade 3 at 72 h post-LTx as graded by the ISHLT.^15^

### Ex vivo lung perfusion

EVLP based on the Toronto protocol was performed as described earlier.^16^, ^17^ As per protocol in our center, marginal donor lungs received thrombolytic treatment with 100.000 IU of urokinase after 1 h of perfusion.

### Filtration setup

The following components were used to create the venovenous parallel filtration system: BioR 02 Plus leukocyte filters (Fresenius Kabi, Bad Homburg, Germany), Stockert S5 roller pump (LivaNova, London, United Kingdom), autologous Blood Reinfusion Bag (Sorin Group, Milan, Italy) and a Cardio Kit (Sorin Group, Milan, Italy). Perfusion fluid was actively withdrawn from the venous return to the blood reinfusion bag. Thereafter, the perfusion fluid passed through four BioR 02 Plus filters with the filtrate directed to the EVLP reservoir (Fig. 1B). A flow rate, shown on the pump unit, was maintained over the parallel filtration system with no depleting effect on the reservoir fluid level.

### LG6 filter

An unused LG6 was incorporated into the EVLP circuit at the end of EVLP. Perfusion with the residual perfusion fluid was continued for 2 h, thus without the donor lungs attached to the circuit. Leukocyte counting was performed every 30 min

### Samples

Perfusate samples were collected from sample ports before the inlet and after the outlet of the LG6 during EVLP at 10 min intervals for the first hour. Thereafter, samples were taken in 30 min intervals. In the case of the intervention group, samples were taken from the outlet of the filtration system at the same intervals. Samples were analyzed for leukocyte count and differentiation into mono- and polymorphonuclear cells (Sysmex XN, Sysmex Corporation, Kobe, Japan). Additional samples were centrifuged and stored at −80 °C until further analysis.

### Biomarkers

The following Enzyme-Linked Immunosorbent Assays (ELISA) kits were used(R&D Systems Europe, Abingdon, UK): Interleukin-1β (IL-1β, DY201), Interleukin-6 (IL-6, DY206), Interleukin-8 (IL-8, DY208), Tumor Necrosis Factor Alpha (TNFα, DY210), Hyaluronan (DY3614), Syndecan-1 (DY2780), Vascular Cell Adhesion Molecule-1 (VCAM-1, DY809), CD14 (DY383), CD38 (DY2404–05), CD86 (DY141–05) and neutrophil elastase (NE, DY9167–05). All kits were validated for use with Steen solution. Adjustments were performed in case of perfusate refreshment or volume changes during EVLP.

### Statistical analysis

Data were tested for normality using the Shapiro-Wilk test and QQ plots. Mann-Whitney U test was performed with unpaired non-normally distributed data and the unpaired T-test for normally distributed data. A paired T-test or a Wilcoxon rank-sum test was used for paired data. The Spearman correlation was utilized for correlation analysis. A p-value < 0.05 (two-tailed) was considered significant.

## Results

A total of 15 bilateral EVLPs were performed, of which 6 were in the control group and 9 in the Intervention group. All donor lungs in the control group and 6/9 were transplanted in the intervention group.

### Declined donor lungs

Three donor lungs in the intervention group were declined for LTx. The first pair of donor lungs (medical indication) had an irreparable preexisting air leak, while lung function during EVLP was sufficient to accept for LTx. These lungs were deflated when they arrived in the hospital, otherwise they would have been transplanted directly. The second pair (medical indication) was declined due to insufficient oxygenation capacity <40 kPa despite recruitment manouvers. The third pair (logistical indication) had borderline-acceptable PO_2_ and was also destined for a complex recipient. Unfortunately, no other recipient was found for these donor lungs. Pathology reported: (1) no abnormalities found, (2) bronchitis possibly in an acute setting and several small pneumonia foci and (3) aspiration in the left lower lobe with signs of diffuse alveolar damage.

### Donor and recipient characteristics

Donor characteristics only showed a significantly higher BMI in the intervention group (Table 1). Recipient and donor lung preservation characteristics were similar between groups (Table 2).

**Table 1:** Donor Characteristics

**Donor**	**Control (n = 6)**	**Intervention (n = 9)**	**p-value**
Age (years)	45.3±25.9	49.7±14.1	0.719
Female % (n)	50 (3)	66.7 (5)	0.838
Height (cm)	177.5±6.89	177.1±11.8	0.944
Weight (kg)	76.58±9.05	85.8±13.3	0.164
BMI (kg/m^2^)	24.2±2.1	27.1±2.1	***0.022***
Predicted TLC (L)	6.7±1.1	6.5±1.4	0.698
DBD/DCD	5/1	3/6	0.066
Smoking history % (n)	16.7 (1)	33.3 (3)	0.610
Last PO_2_ (kPa)	59.1±14.2	47.0±15.9	0.166
Cause of death			
OHCA	-	3	
Trauma	2	3	
Cerebrovascular			
− Bleeding	3	-	
− Stroke	1	-	
Strangulation	-	2	
Euthanasia	-	1	
Thrombolytic therapy EVLP % (n)	33.3 (2)	33.3 (3)	0.999

BMI, Body Mass Index; DBD, Donation after Brain Death; DCD, Donation after Circulatory Death; TLC, Total Lung Capacity; PO_2_, arterial partial oxygen pressure at PEEP 5 cmH_2_O and FiO_2_ 1.0; PE, pulmonary emboli; OHCA, Out Of Hospital Cardiac Arrest. Values are expressed as mean with the standard deviation.

**Table 2:** Recipient Characteristics

	**Control (n = 6)**	**Intervention (n = 6)**	**p-value**
Age (years)	56.5±7.5	56.5±7.7	> 0.99
Female % (n)	50.0 (3)	55.6 (5)	0.399
Height (cm)	176.6±10.8	170.2±10.3	0.317
Weight (kg)	73.5±14.6	71.3±8.7	0.752
BMI (kg/m^2^)	23.3±3.7	24.6±2.6	0.526
LAS	59.0±30.4	36.5±8.5	0.109
FEV (L/s)	1.1±0.7	1.04±0.44	0.404
TLC (L)	6.9±3.4	7.4±2.62	0.812
ECMO per-Tx % (n)	100 (6)	66.7 (4)	0.138
VA-ECMO % (n)	100 (6)	100 (4)	-
ECMO post-TX % (n)	50 (3)	16.7 (1)	0.999
VA-ECMO % (n)	2 (67)	-	-
VV-ECMO % (n)	1 (33)	1 (100)	-
CIT I (min)	238±92.5	290.5±63.4	0.107
EVLP duration (min)	283.2±53.4	275.5±53.7	0.785
CIT II 1st lung (min)	245.2±86.5	276.5±118.6	0.613
CIT II 2nd lung (min)	376.0±115.1	406.8±155.8	0.706
Lung disease			
Emphysema	3	3	
Primary pulmonary hypertension	1	-	
Idiopathic pulmonary fibrosis	2	2	
Sarcoidosis	-	1	
Conversion rate (%)	100	66.7	0.127
ICU stay (days)	3.8±1.7	2.0±1.4	0.730
Mechanical ventilation (days)	3.0±0.7	1.4±0.5	0.556

BMI, Body Mass Index; LAS, Lung Allocation Score; TLC, Total Lung Capacity; ECMO, Extra Corporeal Membrane Oxygenation; VA, Veno-Arterial; VV, Veno-Venous; CIT I, Cold Ischemia Time I (from start in situ donor antegrade flush till start EVLP); CIT II, Cold Ischemia Time II (from start cooling EVLP till in situ recipient reperfusion). Values are expressed as mean with the standard deviation.

### Circulating donor passenger leukocytes

**Figure 2:**
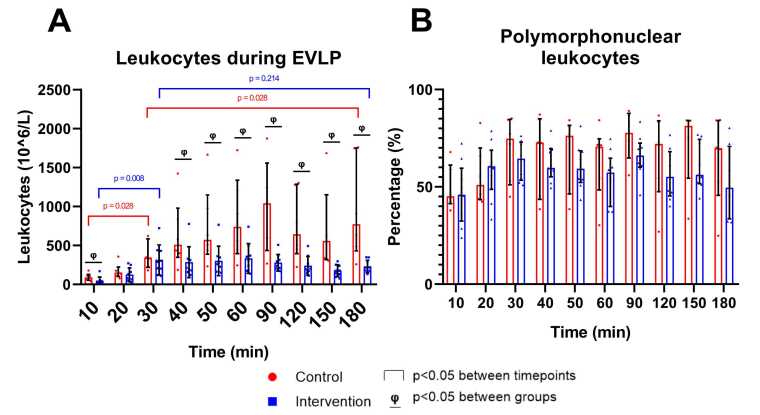
Donor leukocytes during EVLP of the control (red circle) and intervention group (blue square). (A) Circulating donor passenger leukocytes during EVLP. (B) Circulating donor passenger leukocytes expressed in percentage as polymorphonuclear (PMN) leukocytes. Individual datapoints are displayed and values are expressed as median with IQR. Table S1 and S2.

Leukocytes in both groups increased significantly from 10 to 30 min. Subsequently, the number of leukocytes in the control group was significantly higher at 180 min compared to 30 min (p=0.028). In contrast, the intervention group leukocyte count remained stable from 30 to 180 min and was significantly lower than that in the control group (p=0.214)(Figure 2A, table S1). The percentage of polymorphonuclear leukocytes was similar between groups (Figure 2B, table S2).

### Filtration capacity and efficiency

**Figure 3:**
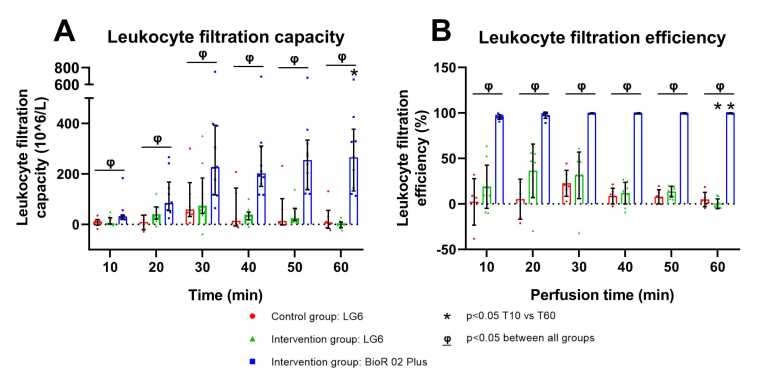
Leukocyte filtration characteristics of the LG6 in the control group (red circle), the LG6 in the intervention group (green triangle) and the BioR 02 plus filters (blue square). (A) Leukocyte filtration capacity, determined by the difference in leukocyte concentration between the in- and outlet of the filters, and (B) Leukocyte filtration efficiency, percentage difference in amount of leukocytes between the in- and outlet of the filters. Individual data points are shown and values expressed as median with IQR. Table S3 and S4.

In both groups, the LG6 showed a significant increase in leukocyte filtration capacity within the first 30 min, after which it decreased to negligible levels at 60 min (Figure 3A, table S3). Noteworthy is that, on occasion, more leukocytes were measured in the outlet than in the inlet. The filtration capacity in the intervention group remained significantly higher at all time points. Also, the filtration efficiency of the intervention group was > 90% at all time points and was significantly higher than that of the LG6. The highest efficiency of the LG6 appears to occur at 30 min (Figure 3B, table S4). Whilst the filtration capacity and efficiency in the intervention group remained stable up to 300 min (not shown). A flow rate between 0.10 and 0.15 L/min could be maintained over the filtration system.

### LG6 efficiency

**Figure 4:**
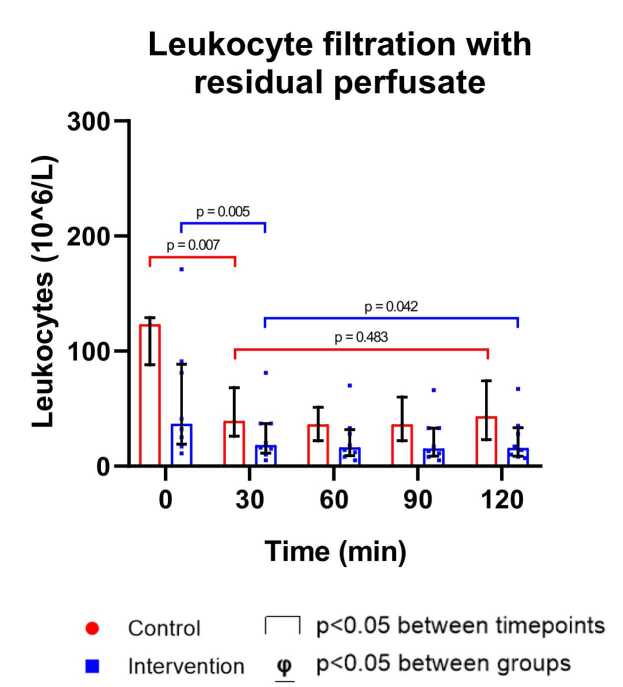
Leukocyte filtration with new unused LG6 filters with the residual EVLP perfusate after completed procedures of both control (red circle, n=3) and Intervention groups (blue square, n=8). Individual datapoints are displayed and values are expressed as median with IQR. Table S5.

An unused LG6 was incorporated into the circuit after 11 EVLP procedures, of which 3 were in the control (302.5 ± 30.4 min) and 8 in the intervention group (274.6 ± 57.7 min). The leukocyte concentration decreased significantly within 30 min (control p=0.007, intervention p=0.005) (Figure 4, table S5). The absolute leukocyte reduction is minimal when compared to during EVLP (Figure 2A).

### Proinflammatory cytokines, endothelium and leukocyte activation

**Figure 5:**
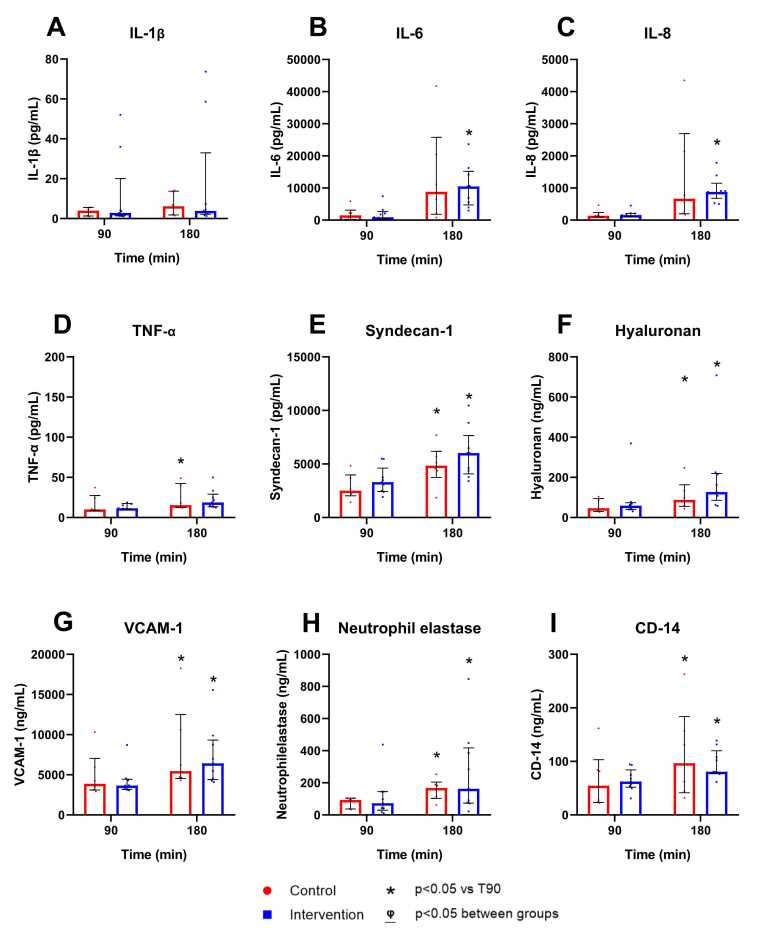
Pro-inflammatory cytokines, endothelial glycocalyx degradation products and endothelial cell activation marker at 90 and 180 min of EVLP, control (red circle) and intervention group (blue square). (A) Interleukin-1β, (B) Interleukin-6 (IL-6; logistical (C) Interleukin-8, (D) Tumor Necrosis Factor-α (E) Syndecan-1, (F) Hyaluronan, (G) Vascular Cell Adhesion Molecule-1 (VCAM-1), (H) Neutrophil elastase and (I) CD-14. Individual datapoints are displayed and values are expressed as median with IQR. Table S6.

The concentration of syndecan-1 (control: p=0.043, intervention: p=0.008), hyaluronan (control: p=0.043, intervention: p=0.043), VCAM-1 (control: p=0.028, intervention: p=0.012), NE (control: p=0.028, intervention: p=0.008) and CD-14 (control: p=0.028, intervention: p=0.008) increased significantly from 90 to 180 min in both groups. No significant differences were seen between groups (Table S6). IL-6 and IL-8 increased significantly in the intervention group (p=0.028 and p=0.028), while TNF-α increased significantly only in the control group (p=0.043). No detectable levels could be measured for both groups in terms of CD38 (< 31.2 pg/mL) and CB86 (< 46.9 pg/mL) (no graphs shown) (Figure 5). The ratio NE/CD-14 did not significantly change over time in the control (T90: 2.3 IQR [0.5–2.5], T180: 2.0 IQR [0.8–4.3], p=0.463) and the intervention group (T90: 1.0 IQR [0.5–2.5], T180: 1.1 IQR [0.6–3.7], p=0.173). At both timepoints the ratios were similar (T90: p=0.556, T180: p=0.724).

### EVLP parameters and short-term clinical outcomes

**Figure 6:**
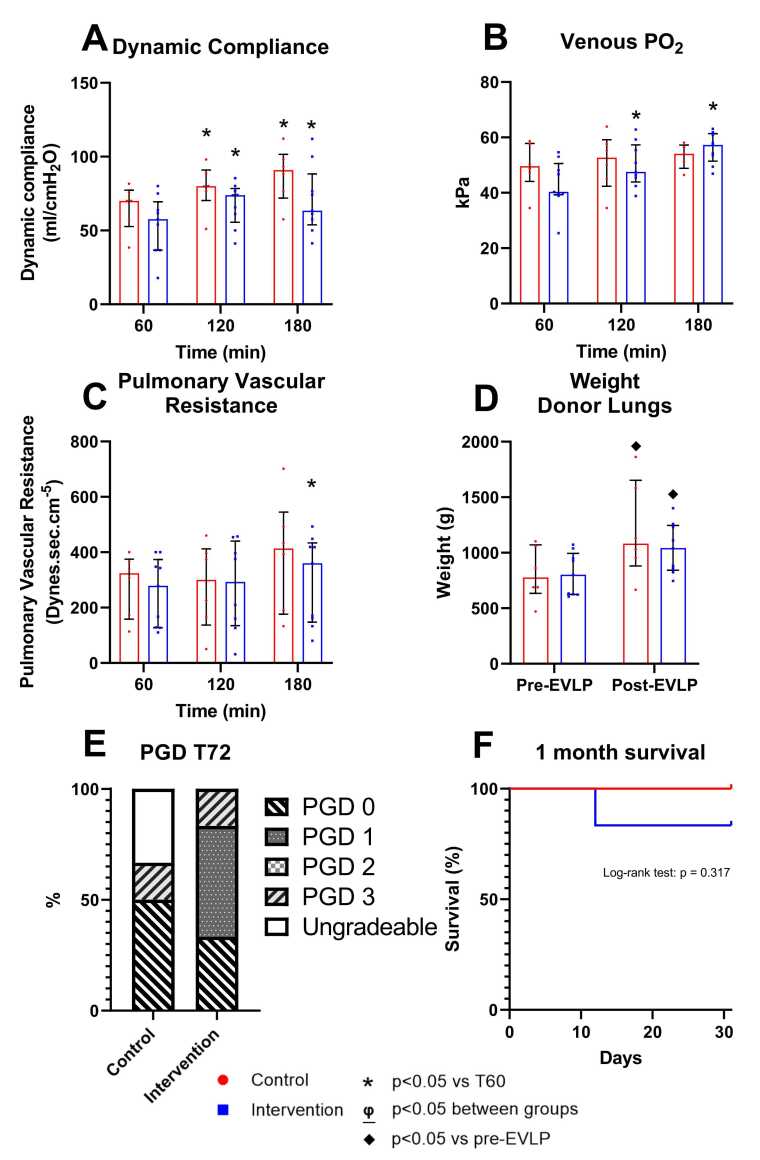
EVLP parameters and clinical outcomes of the control (red circle) and intervention (blue square) group. (A) Dynamic compliance (Cdyn), (B) PO_2_,(C) Pulmonary Vascular Resistance, (D) Donor lung weight post-EVLP as expressed in percentage to weight pre-EVLP, (E) Primary graft dysfunction after 72 h, graded as 0–3 or ungradable according to the ISHLT 2016 PGD guidelines.^15^ Incidence is expressed as percentage and (F) 1 month survival, survival is expressed as percentage. Individual datapoints are displayed and values are expressed as median with IQR. Table S7.

Dynamic compliance (Cdyn) increased significantly in both groups (control: p=0.028, intervention: p=0.015, Figure 6A), whereas the PO_2_ increased significantly only in the Intervention group (control: p=0.050, intervention: p=0.008, Figure 6B). Moreover, pulmonary vascular resistance (PVR) remained comparable between groups throughout EVLP (Figure 6C). Donor lungs in both groups gained significant weight during EVLP (control: p=0.028, intervention p=0.008), with no significant intergroup difference (p=0.113, Figure 6D).

PGD grades at 72 h were similar between the control and intervention group (grade 0: 50.0% vs 33.3%; grade 1: 0% vs 33%; grade 3: 16.7% vs 16.7%, p = 0.476) (Figure 6E). Two recipients in the control group required VA-ECLS for hemodynamic support, resulting in ungradable PGD. All recipients of the control group survived the first month (Figure 6F). One recipient in the intervention group became suddenly acutely unwell on the ward, 12 days after an uncomplicated LTx and ICU stay. Cardiopulmonary Resuscitation for over 1 h was unfortunately unsuccessful. Obduction identified a likely acute cardiac event causing severe pulmonary edema and subsequent respiratory failure (Figure 6F). The ICU stay and duration of mechanical ventilation were similar (Table 2).

### Correlation analysis

No significant correlations were found between the number of circulating leukocytes and the measured biomarkers after 180 min, EVLP parameters (PVR, Cdyn, and PO_2_), PGD grade after 72 h or 1-month survival.

## Discussion

Standard practice for EVLP is to incorporate a leukocyte filter to mitigate the harmful effects of circulating donor passenger leukocytes.^2^, ^18^ We observed continuous leukocyte migration in the perfusate during EVLP, providing a potential target prior to LTx. Although our filtration system efficiently reduced circulating leukocytes compared with the standard LG6, this did not attenuate inflammation, endothelial injury, or improve lung function during EVLP. PGD grade 3 and 1-month survival were comparable between groups. Donor leukocytes can migrate rapidly upon reperfusion and may drive inflammation, IRI and alloreactivity.^2^, ^19^, ^20^ In IRI, monocytes act as early responders, orchestrating neutrophil activation and migration in the delayed phase.^20^, ^21^ During EVLP, substantial non-classical monocytes can mobilize within 30 min^2^ Interestingly, Lee et al. reported the most abundant leukocyte subtype, consistent with our observations. ^1^ Consequently, leukocyte filters are commonly incorporated into the EVLP circuit to limit leukocyte activation, endothelial injury, and inflammatory cascades. ^1–3,12,13^ The LG6 is a widely used inline leukocyte filter; however, its efficacy during EVLP has not been fully established. Clinical EVLP studies suggest that inflammatory cytokines still accumulate despite LG6 use, with maximal leukocyte reduction occurring within 15–30 min and no demonstrated benefit in a porcine EVLP model.^12^, ^22^, ^23^, ^24^, ^25^, ^26^, ^27^, ^28^, ^29^ Notably, LG6 filters failed to remove leukocytes after processing 1 L of porcine blood.^10^ Taken together, these findings suggest that the LG6 may have limited effectiveness during prolonged perfusion, likely due to rapid saturation.^2^, ^3^, ^12^, ^30^

Consistent with our findings, the LG6 achieved significant filtration only within the first 30 min of EVLP and only when perfusion was continued through a new LG6 using the residual perfusate. Although the decrease was significant, the absolute decrease in leukocytes was negligible relative to the total circulating leukocytes (Figure 4). The LG6 filters appear to be associated with increased leukocyte activation, potentially resulting in detachment from the filter, remigration into the donor lungs, and the release of inflammatory cytokines by retained leukocytes.^30^, ^31^ The detachment may explain why leukocyte counts were occasionally higher in the outlet than in the inlet of the LG6. Consequently, we clamped the LG6 after 60 min of EVLP in both groups. This phenomenon was not observed in the intervention group. Additionally, a comparable filter to the BioR 02 plus used in the intervention group showed that trapped leukocytes after rat EVLP did not secrete IL-6.^13^

Contrary to our hypothesis, the significant reduction in circulating leukocytes achieved by our filtration system did not alter biomarker levels or improve lung function. In contrast, a rat EVLP study showed that leukocyte filtration reduced IL-6 levels, attenuated inflammatory gene expression and improved lung function. This study suggested that circulating leukocytes, rather than circulating cytokines, significantly impaired lung function. ^13^ Our results do not show this, possibly because of methodological differences, as laboratory rat lungs differ considerably in size and quality from clinical donor lungs. Interestingly, attenuation of cytokine accumulation during clinical EVLP appears to improve graft function and survival.^22^, ^32^

In addition to inflammatory cytokines, we measured cell-specific markers NE, CD14, CD38, and CD86, reflecting activation of neutrophils, monocytes/macrophages, T cells, and Antigen Presenting Cells (APCs), respectively. Only NE and CD14 were measurable, whereas CD38 and CD86 remained below the ELISA detection limits, suggesting a predominance of activated neutrophils, monocytes, and macrophages. Both NE and CD14 increased significantly during EVLP, but no differences were observed between the control and intervention groups. This may suggest that these circulating donor leukocytes do not directly influence leukocyte activation during EVLP. One hypothesis is that these leukocytes exert limited inflammatory effects or are inactive, rendering filtration ineffective in attenuating inflammation. As CD38 and CD86 are membrane-bound proteins, elevated or detectable levels may reflect cellular disruption like mechanical lysis or fragmentation.^33^, ^34^ Neutrophils and macrophages, given their relatively large size, may render them more susceptible to mechanical forces caused by the closed EVLP circuit, causing cellular injury and accumulation of NE and CD14. Nonetheless, we did not significantly differences in NE/CD14 ratios between groups and over time. Further investigation may clarify the rationale for leukocyte filtration. Additionally, these circulating leukocytes are not transplanted as they remain in the perfusate after EVLP. This may explain the similar short-term clinical outcomes between groups.

Targeting neutrophil activation may be a promising therapeutic strategy. In preclinical models, NE inhibition reduced pro-inflammatory cytokine release, improved lung function and reduced.^35^, ^36^ Elevated soluble CD14 levels in transplant recipients have been associated with a reduced incidence of PGD, possibly through dampening immune activation.^37^ CD38 inhibition, a marker of activated T-cells, has demonstrated endothelial protective effects and improved outcomes in antibody-mediated rejection. ^38^ In our study, CD38 and CD86 were undetectable, suggesting minimal T-cell and APC activation (Figure 2B). Combining therapies that inhibit leukocyte activation during EVLP and reperfusion may further mitigate IRI and PGD development.

Other sources than leukocytes may contribute to inflammation, as the endothelium can also release cytokines.^39^ This is supported by increased endothelial activation marker VCAM-1 in both groups, as well as endothelial glycocalyx shedding(hyaluronan and syndecan-1). Activated leukocytes may adhere to the activated endothelium, making filtration ineffective in removing these adherent cells.^40^ This may explain why inflammatory cytokines and leukocyte activation markers were similar between groups. Other approaches to eliminate and prevent these activated and adhered leukocytes should be investigated.

Interestingly, donor leukocytes seem not to be always detrimental. Donor chimerism has been associated with improved allograft survival, whereas its loss accompanies acute rejection.^41^, ^42^ Moreover, donor leukocyte infusion may reduce rejection and enhance graft survival.^43^, ^44^ Nonetheless, the precise effect of EVLP and donor passenger leukocytes on clinical outcomes and chimerism requires further investigation.

Our primary objective, to provide an alternative strategy to significantly reduce circulating donor passenger leukocytes during EVLP, was achieved. However, the specific effect of the filtration system could not be fully determined because all groups initially used the LG6 filter. Nevertheless, consistent with previous reports, leukocyte removal by a single LG6 filter is likely negligible. Although the filtration system effectively reduced circulating donor leukocytes, its passive-flow design may limit efficiency, allowing partial leukocyte recirculation. An inline filter would be preferable; however, no such devices are currently commercially available. The relatively small group sizes limit statistical power, and larger cohorts would be required to detect differences in PGD. Also, a randomized design may prevent selection bias and a prospective interventional design would be preferred for future studies. We did not investigate whether leukocytes were retained within the filter or elsewhere in the circuit. Such analyses, together with graft tissue sampling and assessment of cell viability, could have provided further insight into leukocyte dynamics and the underlying mechanisms.

## Conclusion

Significantly reducing circulating donor passenger leukocytes did not attenuate inflammatory cytokines, endothelial glycocalyx shedding, endothelial activation or leukocyte activation. Lung function during EVLP remained similar. The precise role of circulating donor passenger leukocytes and their impact on clinical outcomes warrants further investigation.

## Financial disclosure

All authors have no financial disclosures to declare.

## Author Contributions

All authors contributed significantly to this work. Study design: M.H., R.F., M.E. Data collection: M.H. Data analysis and interpretation: M.H., Z.Z., R.F., E.V., T.G., C.W., H.L., M.E. Manuscript writing: MH. All authors reviewed and provided critical feedback, contributing to the final manuscript.

## Declaration of Competing Interest

The authors declare that they have no known competing financial interests or personal relationships that could have appeared to influence the work reported in this paper. H.L. is the Chief Scientific Officer for 34Lives, a Public Benefit Organisation. M.E. holds a patent with XVIVO perfusion.
