# Supplementary material for: Eliminating circulating donor passenger leukocytes during clinical ex vivo lung perfusion does not attenuate inflammation
Source: JHLT Open. 2026 Jul 6;13:100619. doi: 10.1016/j.jhlto.2026.100619 (PMC13396920; doi:10.1016/j.jhlto.2026.100619)
Supplement: Supplementary file 1 — Supplementary material [file mmc1.docx]

**Supplementary materials**

Eliminating Circulating Donor Passenger Leukocytes During Clinical Ex Vivo Lung Perfusion Does Not Attenuate Inflammation

M.A. Hu MD^1^ Orcid# 0000-0002-7355-099X

Z.L. Zhang MD^1^ Orcid# 0000-0003-2137-7555

R.F. Hoffmann PhD^1^ Orcid# 0000-0003-2473-4124

C.T. Gan MD PhD^2^ Orcid# 0000-0002-1310-4189

E.A.M. Verschuuren MD PhD^2^ Orcid# 0000-0002-6807-6744

C. Van De Wauwer MD PhD^1^ Orcid# [0000-0002-0886-4917](http://orcid.org/0000-0002-0886-4917)

H.G.D. Leuvenink PhD^3^ Orcid# 0000-0001-5036-2999

M.E. Erasmus MD PhD^1^ Orcid# 0000-0002- 6234-2976

*^1^Department of Cardiothoracic Surgery. University Medical Center Groningen. Groningen. Netherlands*

*^2^ Department of Pulmonary Diseases and Lung Transplantation. University Medical Center Groningen. Groningen. Netherlands*

*^3^ Department of Surgery. University Medical Center Groningen. Groningen. Netherlands*

Table of contents

Supplementary tables

Table S13

Table S23

Table S34

Table S44

Table S54

Table S65

Table S76

Table S1. Donor leukocytes (10^6^/L) during EVLP of the control and intervention group. Values are expressed as median with IQR.

|  | **Donor leukocytes (10^6^/L) during EVLP** | | | |  |
| --- | --- | --- | --- | --- | --- |
|  | **Control** | | **Intervention** | |  |
| Time (min) | **Median** | **IQR** | **Median** | **IQR** | **P-value** |
| 10 | 86.02 | 52.87 - 127.64 | 30.08 | 22.26 - 48.33 | ***0.018*** |
| 20 | 149.38 | 101.75 - 221.95 | 80.64 | 56.28 - 205.35 | 0.289 |
| 30 | 339.96 | 220.46 - 585.12 | 289.52 | 161.50 - 434.42 | 0.409 |
| 40 | 506.50 | 347.30 - 980.95 | 214.32 | 168.24 - 321.72 | ***0.034*** |
| 50 | 570.48 | 387.09 - 1148.78 | 259.44 | 162.60 - 354.30 | ***0.025*** |
| 60 | 735.81 | 397.44 - 1337.61 | 307.40 | 176.54 - 431.88 | ***0.025*** |
| 90 | 1039.36 | 434.13 - 1558.46 | 227.36 | 202.02 - 339.52 | ***0.014*** |
| 120 | 655.57 | 395.27 - 1285.49 | 199.10 | 159.12 - 318.13 | ***0.007*** |
| 150 | 554.17 | 320.63 - 1151.09 | 176.58 | 107.34 - 245.35 | ***0.003*** |
| 180 | 770.63 | 430.63 - 1750.79 | 215.61 | 164.45 - 318.12 | ***0.005*** |
|  |  |  |  |  |  |

Table S2. Circulating donor passenger leukocytes expressed in percentage as polymorphonuclear (PMN) leukocytes during EVLP.

|  | **Polymorphonuclear leukocytes (%)** | | | | |
| --- | --- | --- | --- | --- | --- |
|  | **Control** | | **Intervention** | |  |
| Time (min | **Median** | **IQR** | **Median** | **IQR** | **P-value** |
| 10 | 45.04 | 41.37 - 61.20 | 45.76 | 32.47 - 59.53 | 0.814 |
| 20 | 50.88 | 43.40 - 69.96 | 60.42 | 48.74 - 68.79 | 0.724 |
| 30 | 74.64 | 51.01 - 84.60 | 64.35 | 53.38 - 72.87 | 0.289 |
| 40 | 72.62 | 43.62 - 84.94 | 59.66 | 55.17 - 69.57 | 0.409 |
| 50 | 76.14 | 46.40 - 81.59 | 59.22 | 52.26 - 68.50 | 0.409 |
| 60 | 70.58 | 48.48 - 74.71 | 57.24 | 39.98 - 64.72 | 0.239 |
| 90 | 77.63 | 64.80 - 87.77 | 65.93 | 60.16 - 72.48 | 0.125 |
| 120 | 71.93 | 47.57 - 83.99 | 55.06 | 45.38 - 68.13 | 0.195 |
| 150 | 81.21 | 54.42 - 84.03 | 56.00 | 51.66 - 74.40 | 0.125 |
| 180 | 69.76 | 45.62 - 84.15 | 49.47 | 33.52 - 70.81 | 0.289 |

Table S3. Leukocyte filtration capacity (10^6^/L), determined by the difference in leukocyte concentration between the in- and outlet of the filters. Values are expressed as median with IQR.

|  | **Leukocyte filtration capacity (10^6^/L)** | | | | | | |
| --- | --- | --- | --- | --- | --- | --- | --- |
|  | **Control: LG6** | | **Intervention: LG6** | | **Intervention: BioR 02 Plus** | |  |
| Time (min) | **Median** | **IQR** | **Median** | **IQR** | **Median** | **IQR** | **P-value** |
| 10 | 10.00 | -3.00 - 19.25 | 31.00 | 20.50 – 44.00 | 3.50 | 1.00 - 23.5 | ***0.015*** |
| 20 | 8.50 | -20.75 - 36.25 | 81.00 | 52.50 - 192.50 | 40.00 | 21.00 – 69.00 | ***0.004*** |
| 30 | 58.50 | 31.50 - 165.75 | 228.00 | 148.00 – 394.00 | 69.50 | 46.50 - 154.50 | ***0.012*** |
| 40 | 13.00 | -6.75 – 144.00 | 210.00 | 152.50 - 314.50 | 36.00 | 1.50 - 43.25 | ***0.001*** |
| 50 | 12.50 | -2.00 - 102.25 | 238.00 | 132.00 - 347.50 | 24.50 | 16.75 - 62.50 | ***0.001*** |
| 60 | 7.50 | -15.50 – 56.00 | 265.00 | 132.00 – 377.00 | -3.00 | -13.00 - 9.50 | ***0.000*** |

Table S4. Leukocyte filtration efficiency (%), percentage difference in amount of leukocytes between the in- and outlet of the filters. Individual data points are shown and values expressed as median with IQR. Values are expressed as median with IQR.

|  | **Leukocyte filtration efficiency (%)** | | | | | | |
| --- | --- | --- | --- | --- | --- | --- | --- |
|  | **Control: LG6** | | **Intervention: LG6** | | **Intervention: BioR 02 Plus** | |  |
| Time (min) | **Median** | **IQR** | **Median** | **IQR** | **Median** | **IQR** | **P-value** |
| 10 | 7.86 | -31.24 - 15.64 | 13.85 | 96.97 - 13.85 | 95.45 | 4.76 - 33.11 | ***< 0.001*** |
| 20 | 11.34 | -16.62 - 28.46 | 47.30 | 99.61 - 47.29 | 98.78 | 19.51 - 55.69 | ***< 0.001*** |
| 30 | 24.67 | 12.47 - 37.23 | 32.07 | 99.75 - 32.07 | 99.16 | 25.61 - 47.68 | ***< 0.001*** |
| 40 | 5.72 | -2.38 - 15.66 | 13.33 | 99.76 - 13.33 | 99.39 | 2.02 - 21.49 | ***< 0.001*** |
| 50 | 7.66 | 3.36 - 13.33 | 14.93 | 99.85 - 14.93 | 99.65 | 7.77 - 19.4 | ***< 0.001*** |
| 60 | 2.08 | -4.76 - 4.56 | -2.63 | 99.73 - -2.63 | 99.70 | -3.58 - 5.21 | ***< 0.001*** |

Table S5.Leukocyte filtration (10^6^/L) with new unused LG6 filters with the residual EVLP perfusate after completed procedures of both control (n=3) and intervention groups (n=8)Values are expressed as median with IQR.

|  | **Leukocyte filtration LG6 filters with the residual EVLP perfusate** | | | | |
| --- | --- | --- | --- | --- | --- |
|  | **Control (n=3)** | | **Intervention (n=8)** | |  |
| Time (min) | **Median** | **IQR** | **Median** | **IQR** | **P-value** |
| 0 | 123.00 | NA | 36.50 | 19.00 - 88.50 | 0.102 |
| 30 | 39.00 | NA | 18.00 | 11.25 – 37.00 | 0.152 |
| 60 | 36.00 | NA | 16.00 | 9.00 - 31.75 | 0.153 |
| 90 | 36.00 | NA | 15.00 | 8.75 – 33.00 | 0.152 |
| 180 | 43.00 | NA | 15.50 | 8.50 - 33.25 | 0.102 |

Table S6. Comparison of the measured biomarkers of the donor lungs in the control (n=6) and intervention (n=9) group during EVLP at 90 and 180 min of EVLP and the delta value (90-180 min). Values are expressed as median with IQR.

|  | **Control** | | **Intervention** | |  |
| --- | --- | --- | --- | --- | --- |
|  | **Median** | **IQR** | **Median** | **IQR** | **P-value** |
| **IL-1β T90** | 3.94 | 1.33 - 5.58 | 2.80 | 1.34 - 20.06 | 0.814 |
| **IL-1β T180** | 6.17 | 1.86 - 13.76 | 3.86 | 2.15 - 32.98 | 0.814 |
| **Δ IL-1β** | 1.76 | 0.53 - 8.36 | 1.61 | 0.25 - 12.5 | 0.814 |
| **IL-6 T90** | 1473.40 | 214.14 - 3097.79 | 906.40 | 355.01 - 2650.07 | 0.906 |
| **IL-6 T180** | 8813.52 | 1827.7 - 25758.5 | 10472.02 | 4736.35 - 15198.46 | 0.906 |
| **Δ IL-6** | 7320.50 | 1632 - 22690.47 | 8546.04 | 4406.63 - 13221.32 | 1.000 |
| **IL-8 T90** | 134.55 | 99.6 - 236.58 | 155.60 | 111.01 - 209.82 | 0.556 |
| **IL-8T180** | 664.74 | 199.22 - 2693.59 | 878.66 | 681.43 - 1149.27 | 0.556 |
| **Δ IL-8** | 530.20 | 99.62 - 2457 | 723.06 | 545.6 - 966.2 | 0.556 |
| **TNF-α T90** | 10.04 | 9.06 - 27.38 | 11.53 | 10.46 - 17.47 | 0.409 |
| **TNF-α T180** | 15.48 | 12.4 - 42.41 | 18.83 | 13.37 - 29.13 | 0.480 |
| **Δ TNF-α** | 6.06 | 3.03 - 12.75 | 3.45 | 2.6 - 11.66 | 0.637 |
| **Syndecan-1 T90** | 2505.97 | 2031.56 - 3982.42 | 3311.54 | 2436.58 - 4612.69 | 0.289 |
| **Syndecan-1 T180** | 4842.36 | 3744.49 - 6183.43 | 6013.03 | 4077.67 - 7660.96 | 0.480 |
| **Δ Syndecan-1** | 2171.06 | 1595.54 - 2566.4 | 2528.96 | 1331.1 - 3443.45 | 0.480 |
| **Hyaluronan T90** | 46.31 | 30.3 - 94.15 | 59.10 | 40.2 - 73.75 | 0.739 |
| **Hyaluronan T180** | 87.55 | 55.83 - 162.79 | 126.78 | 85.65 - 219.8 | 0.289 |
| **Δ Hyaluronan** | 50.00 | 37.2 - 98.67 | 78.84 | 37.9 - 146 | 0.317 |
| **VCAM-1 T90** | 3869.56 | 3103.59 - 7039.93 | 3657.38 | 3188.97 - 4445.13 | 0.814 |
| **VCAM-1 T180** | 5463.88 | 4548.66 - 12510.74 | 6420.45 | 4402.05 - 9321.21 | 0.814 |
| **Δ VCAM-1** | 1843.87 | 1356.62 - 5470.81 | 2949.35 | 1026.17 - 4876.08 | 0.906 |
| **Neutrophil elastase T90** | 77.74 | 36.98 - 104.35 | 112.57 | 29.5 - 145.21 | 0.906 |
| **Neutrophil elastase T180** | 159.32 | 102.62 - 204.67 | 265.16 | 73.67 - 416.93 | 0.724 |
| **Δ Neutrophil elastase** | 81.58 | 52.29 - 102.86 | 152.59 | 40.15 - 295.18 | 0.556 |
| **CD-14 T90** | 66.76 | 23.37 - 103.17 | 64.97 | 51.76 - 84.08 | 0.637 |
| **CD-14 T180** | 114.92 | 41.33 - 183.58 | 95.46 | 78.04 - 119.88 | 0.906 |
| **Δ CD-14** | 48.16 | 15.07 - 80.42 | 30.49 | 22.54 - 35.68 | 0.346 |
|  |  |  |  |  |  |
|  |  |  |  |  |  |
|  |  |  |  |  |  |
|  |  |  |  |  |  |
|  |  |  |  |  |  |
|  |  |  | IQR. Interquartile Range; IL-1. Interleukin-1; IL-6. Interleukin-6; IL-8. Interleukin-8; TNF-α. Tumor Necrosis Factor-α; VCAM-1. Vascular Cellular Adhesion Molecule-1; CD-14. Cluster of differentation 14; T90. 90 min of EVLP; T180. 180 min of EVLP. |  |  |
|  |  |  |  |  |  |
|  |  |  |  |  |  |

Table S7. EVLP parameters of the control and intervention group. Values are expressed as median with IQR.

|  | **Control** | | **Intervention** | |  |
| --- | --- | --- | --- | --- | --- |
| **EVLP Parameter** | **Median** | **IQR** | **Median** | **IQR** | **P-value** |
| PVR (Dynes.sec.cm^-5^) 60 min | 323.66 | 158.31 - 375 | 177.78 | 127.75 - 373.91 | 0.554 |
| PVR (Dynes.sec.cm^-5^) 120 min | 372.88 | 196.20 - 392.89 | 261.23 | 135.02 - 391.19 | 0.558 |
| PVR (Dynes.sec.cm^-5^) 180 min | 412.75 | 176.05 - 545.11 | 265.19 | 147.64 - 433.45 | 0.346 |
| CDyn (mL/cmH_2_O) 60 min | 69.94 | 52.73 - 77.31 | 57.50 | 36.68 - 69.38 | 0.175 |
| CDyn (mL/cmH_2_O) 120 min | 79.93 | 70.28 - 90.88 | 73.86 | 55.61 - 78.38 | 0.099 |
| CDyn (mL/cmH_2_O) 180 min | 90.83 | 71.88 - 101.55 | 63.33 | 53.78 - 88.33 | 0.195 |
| PO_2_ (kPa) 60 min | 49.55 | 44.10 - 57.85 | 40.30 | 39.30 - 50.55 | 0.157 |
| PO_2_ (kPa) 120 min | 52.65 | 42.38 - 59.18 | 47.50 | 43.90 - 57.30 | 0.814 |
| PO_2_ (kPa) 180 min | 54.00 | 48.80 - 57.25 | 57.20 | 51.40 - 61.35 | 0.317 |

*Pulmonary Vascular Resistance (PVR), Dynamic compliance (Cdyn) and venous PO_2_.*
